# A Case of Post-traumatic Persistent Nasal Pain

**DOI:** 10.3389/fneur.2019.01409

**Published:** 2020-02-03

**Authors:** Marta Puma, Barbara Petolicchio, Alessandro Viganò, Ilaria Maestrini, Vittorio Di Piero

**Affiliations:** ^1^Department of Human Neurosciences, Sapienza University of Rome, Rome, Italy; ^2^“Enzo Borzomati” Pain Medicine Unit–University Hospital Policlinico Umberto I, Rome, Italy; ^3^IRCCS Fondazione Don Carlo Gnocchi, Milan, Italy

**Keywords:** headache, intranasal steroids, *radix nasi*, post-traumatic neuropathy, migraine

## Abstract

Nasal pain is a complex and misdiagnosed entity with various peripheral and central etiologies. It is often a bothersome, treatment-resistant condition and could be spontaneous or post-traumatic. Although pain usually occurs immediately after injury, rarely it may occur long after the nasal trauma. Due to its rarity, successful treatment is difficult because of the lack of standardized options. According to our experience, an effective therapeutic approach should take into account both local inflammatory factors and aberrant nerve modulations. We describe the case of a young woman complaining of a delayed onset persistent nasal pain occurring 1 month after facial trauma and who has improved with the addition of intranasal steroids to first-line neuropathic pain treatment.

## Background

Nasal pain is an intricate and fairly rare condition that can be caused by various central and peripheral mechanisms. Correct diagnosis and classification are generally not straightforward for clinical and pathophysiological reasons.

Firstly, the nasal pyramid is densely innervated by several terminal branches of the ophthalmic and maxillary divisions of the trigeminal nerve. Specifically, the nasociliary nerve, a branch of the ophthalmic nerve, divides into the infratrochlear nerve, which supplies the lateral portion of the *radix nasi* and the proximal portion of the nasal dorsum, and the anterior ethmoidal nerve, which gives rise to the external nasal nerve, innervating the distal portion of the nasal dorsum, *ala nasi* and *apex nasi*. In addition, the maxillary nerve gives origin to the infraorbital nerve, which supplies the lateral portion of the *ala nasi* and the anterior superior alveolar nerve for the *columella* (1, 2).

Clinically, nasal pain can have different or even mixed features. For instance, it may either be sharp or dull, paroxysmal or constant, short or long lasting, or deep or superficial. Pain may present alone or with neuropathic negative or positive symptoms. It can occur spontaneously or be post-traumatic, originating immediately or long after a traumatic event, even of a mild degree. Of course, in the differential diagnosis of these painful forms, trigeminal autonomic cephalalgias (TACs) should be excluded.

From the therapeutic point of view, although not common, it can be very bothersome and treatment-resistant, also requiring invasive procedures.

Traumatic nasal pain can affect several nerves at the same time, often causing a kind of pain that is not limited to a single nerve territory and hard to depict. The external nasal nerve is often involved, even for mild trauma, since it runs under the nasal bones and then emerges at the junction between the bone and the cartilage nasal portion; thus, in its superficial course, it is at risk of being entrapped and rubbed between the mobile cartilage and the fixed bone (1, 3, 4).

In literature, a few cases of post-traumatic nasal pain syndrome have been described (1, 2).

Here, we report the case of a young woman suffering from a nasal pain that occurred 1 month after a facial trauma.

## Case Presentation

A 34-year-old woman consulted our outpatient clinic because of a severe persistent right nasal and periorbital pain.

Four months before, she slammed her nasal area against the corner of a closet. The patient did not report any fractures of facial bones, skin injuries, or other complications, so she barely recalled the trauma during the medical interview. The trauma caused an immediate slight pain in the nasal pyramid. She was treated with topical anti-inflammatory therapy and ice-packing with complete pain resolution in a week.

A month later, she developed pain to the right *radix nasi* and the medial ipsilateral periorbital region. The pain was daily, persistent and continuous, and of severe intensity and stabbing/burning quality. The patient did not report any associated autonomic or positive/negative neuropathic symptoms (e.g., hyperalgesia/allodynia, hypoesthesia/hypoalgesia).

In her past medical history, there was episodic migraine without aura since puberty and no other systemic or neuropsychiatric diseases. Physical and neurological examinations were normal. Varicella Zoster Virus and Herpes Simplex Virus infections were excluded with blood test. She underwent neuroimaging (MRI and facial bones CT), which showed a deviation of nasal septum; however, the ENT specialist did not consider it so important to require surgery. Furthermore, no dental causes were detected.

She was taking pregabalin 75 mg twice a day since for a month, with only partial pain relief. We decided to add amitriptyline 10 mg once a day at bedtime.

At a month follow-up visit, the patient reports a slight improvement, but she continued to complain nasal pain, localized to the right nostril *radix*.

In the lack of clear signs of neuropathy and to avoid sedation in a young patient, rather than increasing drugs for neuropathic pain, we preferred to add intranasal fluticasone spray 50 μg twice a day. The patient responded very well to this combination of drugs; in fact, the pain disappeared within a month.

After another month, she gradually discontinued both systemic and topical therapy.

At 6 months follow-up, the patient still reported a complete resolution of symptoms.

An informed written consent was obtained from the patient for the publication of this case report.

## Discussion and Conclusions

The differential diagnosis of nasal pain may be due to many etiologies, either from spontaneous or traumatic causes or from a local or central nature. In our case, the traumatic cause seems to be the most probable, because the pain developed in the region of the face that is previously involved in a minor facial trauma.

The pain involved the nasal portion of the skin provided by the infratrochlear and anterior ethmoid nerves; the painful area therefore did not show the typical topography of a non-traumatic neuralgia that affects one of the terminal branches of the trigeminal nerve, e.g., lacrimal neuralgia, external nasal, infratrochlear, and supraorbital neuralgia (4–6).

In our case, pain started 1 month after the injury. This is not uncommon since, after trauma, nasal pain can start either immediately or after a few weeks/months. The delayed onset of symptoms may be due to a local fibrosis process or to the formation of a neuroma secondary to trauma (1).

In line with most of the cases described in the literature (2, 3), pain was continuous and severe in intensity.

Among different alternatives diagnoses, a possible neuralgic type of nasal pain was excluded because the pain was continuous and not aggravated by tactile stimulus. Although the patient had a diagnosis of migraine without aura, pain had no allodynic features; on the contrary, the low frequency of her migraine attacks does not suggest a significant sensitization process (7). TACs or nasociliary neuralgia were excluded for the pain features and the absence of autonomic symptoms. Also, we excluded painful post-traumatic trigeminal neuropathy (PPTTN—ICHD-3 code 13.1.2.3), since the patient never reported the positive or negative signs of neuropathy (e.g., hyperalgesia, hypoesthesia) that are needed to make this diagnosis.

Considering pain characteristics, our case could be diagnosed as persistent idiopathic facial pain (PIFP-ICHD-3 code 13.12, Table 1) (8). Some authors argue that the differential diagnosis between PIFP and PPTTN could be difficult or arbitrary (9) if we wish to interpret these two pathologies as a continuous clinical spectrum. In favor of PIFP, there are (i) the young age and the sex of the patient (a woman between 30 and 40 years) (10); (ii) the slight entity of the trauma; and (iii) the characteristics of the initial pain relatively poorly defined, suggesting that whatever the cause, the traumatic noxa had resolved at the time the pain started. Finally, our patient had a negative neurological exam and a history of migraine. Indeed, a chronic painful condition is commonly observed in subjects presenting a PIFP (10).

**Table 1:** Diagnostic criteria for PIFP according to ICHD-3.

**13.12 Persistent idiopathic facial pain (PIFP)**
A. Facial and/or oral pain fulfilling criteria B and C B. Recurring daily for >2 h/day for >3 months C. Pain has both of the following characteristics: 1. Poorly localized and not following the distribution of a peripheral nerve 2. Dull, aching, or nagging quality D. Clinical neurological examination is normal E. A dental cause has been excluded by appropriate investigations F. Not better accounted for by another ICHD-3 diagnosis

From a practical point of view, a correct diagnostic assessment (PIFP rather than PPTTN) has allowed us to introduce an otherwise unconventional treatment in post-traumatic neuralgia, e.g., local steroids (11). In our case, the addition of topical administration of corticosteroids has led to a complete pain resolution, and after 9 months, the patients discontinued all drugs. This result was relatively unexpected since prognosis of PIFP is generally poor.

Steroids are prescribed only occasionally in PIFP since about 14.5% of patients used steroids, either systemically or topically (11). Furthermore, patients with PIFP suffer a diagnostic delay on average of 19.3/11.1 months (11); conversely, our patient was treated with steroid 5 months after the onset of symptoms. Therefore, it is possible that the relatively early administration of the steroid may have contributed to the therapeutic success. Being the patient still under amitriptyline and pregabalin, it is not easy to disentangle the therapeutic role of local steroid from that of the other two medications. Both these latter drugs still has margin to increase, being underdosed, but we preferred to add steroid in order to avoid sedation.

From a pathophysiologic point of view, considering the previous history of trauma and the good (even long-term) response to steroids, it is possible that inflammation has played a role in determining the symptomatology. While on the one hand a possible role of local inflammation as the preferred target of intranasal steroids can only be hypothesized, on the other hand, it is certain that the use of the latter has prompted a rapid resolution of a generally persistent pain such as PIFP.

This case highlights the difficulty in the classification and treatment of post-traumatic nasal pain, reinforcing the concept that different mechanisms, both neuropathic and non-neuropathic, can contribute to the persistence of pain. Given the low number of cases reported in the literature, the treatment of these nasal pain syndromes is not standardized and often customized on a case-by-case basis. However, our case suggests that combination of drugs that act on different components of pain might be worth to be tested. Finally, topical steroids could be considered as an early therapeutic option in order to delay, or perhaps avoid, possible invasive procedures.

## Data Availability Statement

The datasets generated for this study are available on request to the corresponding author.

## Ethics Statement

Ethical review and approval was not required for the study on human participants in accordance with the local legislation and institutional requirements. The patients/participants provided their written informed consent to participate in this study. Written informed consent was obtained from the individual(s) for the publication of any potentially identifiable images or data included in this article.

## Author Contributions

MP: manuscript drafting. BP, AV, IM, and VD: manuscript revision. All authors read and approved the final manuscript.

### Conflict of Interest

The authors declare that the research was conducted in the absence of any commercial or financial relationships that could be construed as a potential conflict of interest.
