# Corrigendum: A Case of Post-traumatic Persistent Nasal Pain

**DOI:** 10.3389/fneur.2020.636634

**Published:** 2021-01-05

**Authors:** Marta Puma, Barbara Petolicchio, Alessandro Viganò, Ilaria Maestrini, Vittorio Di Piero

**Affiliations:** ^1^Department of Human Neurosciences, Sapienza University of Rome, Rome, Italy; ^2^“Enzo Borzomati” Pain Medicine Unit–University Hospital Policlinico Umberto I, Rome, Italy; ^3^IRCCS Fondazione Don Carlo Gnocchi, Milan, Italy

**Keywords:** headache, intranasal steroids, *radix nasi*, post-traumatic neuropathy, migraine

In the published article, there was an error regarding the affiliation for Alessandro Viganò.

Instead of affiliation 1 “Department of Human Neurosciences, Sapienza University of Rome, Rome, Italy,” it should be affiliation 3 “IRCCS Fondazione Don Carlo Gnocchi, Milan, Italy.”

The authors apologize for this error and state that this does not change the scientific conclusions of the article in any way. The original article has been updated.

